# Personal

**Published:** 1914-06

**Authors:** 


### PERSONAL.

Announcement of removal, travel, and other matters of interest are requested. Please report errors in the listing of any physician in the State and other directories, that we may co-operate with the State Society in securing a correct list.

Dr. Clayton M. Brown, of Buffalo, announces the removal of his office and residence to 510 Delaware Avenue.

Dr. William T. Getman, of Buffalo, has moved his office to 469 Franklin Street, his residence will be at 428 Richmond Avenue.

Dr. Edward H. Mehl, who has recently returned from Vienna, will be associated with his brother, Dr. William M. Mehl, of Buffalo, at 71 East Genesee Street and will devote his attention to the eye, ear, nose and throat.

Dr. L. G. Hanley, of Buffalo, returned, the middle of May, from a tour through New England, and a visit to his alma mater, Yale University.

Dr. M. Axford, of Buffalo, has moved to 351 Lafayette Avenue.

Dr. W. H. Baker has moved his office from 203 D. S. Morgan Bldg. to Williamsville, his residence.

The following physicians have been appointed members of a local Committee of Arrangement for the 1915 meeting of the State Society: Drs. A. T. Lytle, Arthur G. Bennett, Edith R. Hatch, Lesser Kaufmann, Julius Richter, E. A. Sharp, Carl G. Leo-Wolf, and A. L. Benedict.

Dr. John Ragone, of Buffalo, has returned from Europe.

Dr. Carl G. Leo-Wolf, formerly of Niagara Falls, has returned from Europe and will be located at 481 Franklin Street, Buffalo, limiting his practice to Diseases of Children.

Dr. Prescott Le Breton, of Buffalo, has moved his residence to 15 Manchester Place.

Dr. George E. Musgrove, of Niagara Falls, Ont., has been nominated to the Provincial legislature, by the conservatives.

Dr. A. A. Thibaudeau, of Buffalo, has moved to 23 Irving place.

---

Dr. Thomas S. Blair, of Harrisburg, editor of the Medical Council, has just returned from an extensive western trip. On his way home he stopped in Buffalo and afforded us the opportunity of renewing an old friendship formed at the University of Michigan.

